# Author Correction: Detecting protein and post-translational modifications in single cells with iDentification and qUantification sEparaTion (DUET)

**DOI:** 10.1038/s42003-020-01195-7

**Published:** 2020-08-18

**Authors:** Yandong Zhang, Changho Sohn, Seoyeon Lee, Heejeong Ahn, Jinyoung Seo, Junyue Cao, Long Cai

**Affiliations:** Division of Biology and Biological Engineering, California Institute of Technology, Pasadena, CA USA

Correction to: *Communications Biology* 10.1038/s42003-020-01132-8, published online 3 August 2020.

In the original published version of the article, co-author Seoyeon Lee’s name was incorrectly spelled as Seoyoen Lee. The error has been corrected in the HTML and PDF versions of the article.

