# Supplementary material for: Detecting protein and post-translational modifications in single cells with iDentification and qUantification sEparaTion (DUET)
Source: Commun Biol. 2020 Aug 3;3:420. doi: 10.1038/s42003-020-01132-8 (PMC7400673; doi:10.1038/s42003-020-01132-8)
Supplement: Supplementary file 1 — Supplementary Information [file 42003_2020_1132_MOESM1_ESM.pdf]

Supporting information for

Detecting protein and post-translational modifications in single cells with iDentification and qUantification sEparaTion (DUET)

Table of Contents:

I Supplementary Figure S1 to S7
II Reference

I Supplementary Figures

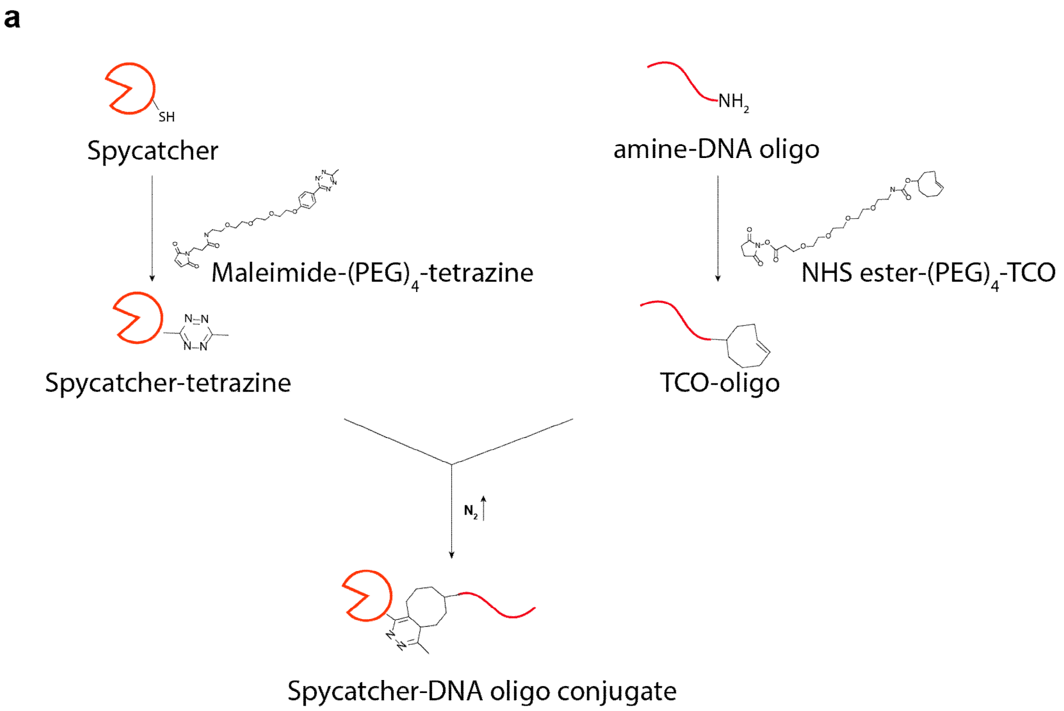

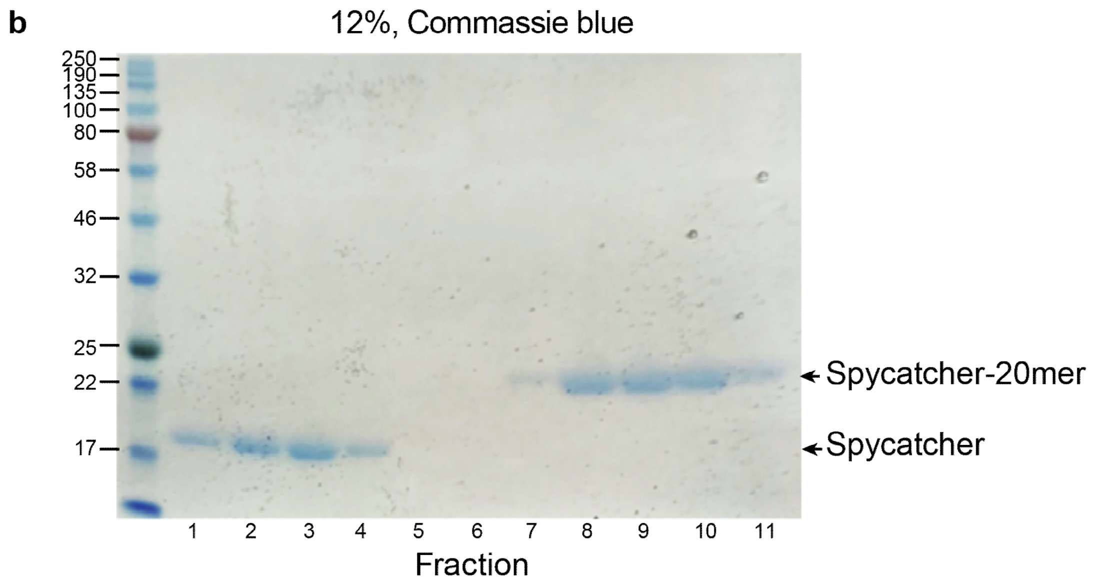

Supplementary Figure 1. Spycatcher-DNA oligo conjugate. a) Strategy for spycatcher-oligo conjugate synthesis. Spycatcher with a cysteine at the C-terminal region was reacted with the maleimide-$(PEG)_4$-methyltetrazine to generate spycatcher-methyltetrazine. 5' amine-modified oligo was reacted with NHS ester-$(PEG)_4$-TCO to generate TCO-oligo. The spycatcher-methyltetrazine and TCO-oligo were then conjugated together via click chemistry. b) The gel electrophoresis results for spycatcher-oligo (20mer) purification using ion-exchange chromatography. The numbers are the different fractions from ion-exchange chromatography. Spycatcher-DNA oligo conjugate(spycatcher-20mer) is separated from the leftover spycatcher.

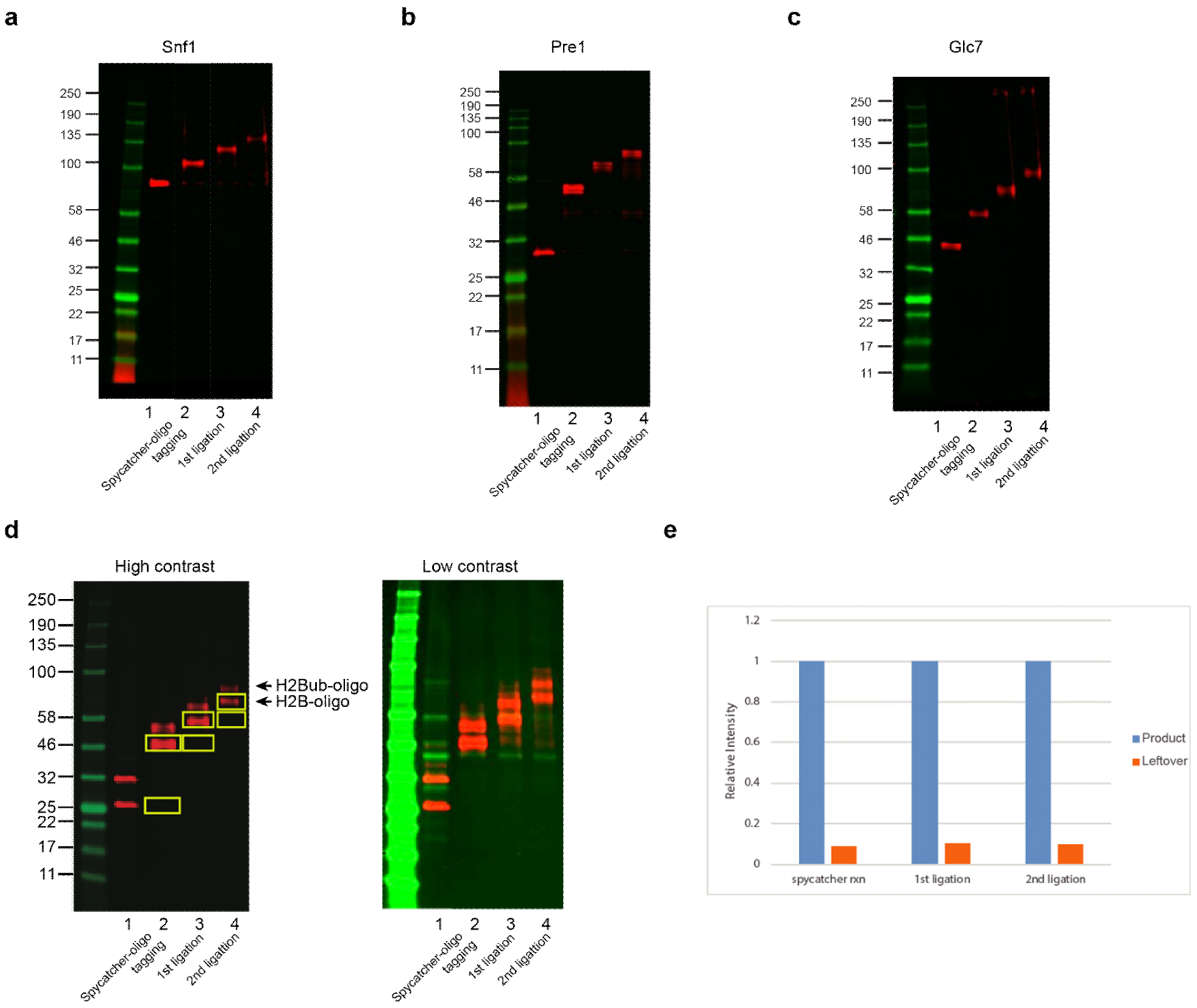

Supplementary Figure 2. Western blot analysis of targeted proteins after spycatcher reaction, the first ligation, and the second ligation. a) The western blot images of Snf1. The red fluorescence corresponds to Snf1 and the green fluorescence corresponds to ladder. The Snf1 bands migrated slower after reactions, indicating successful reactions. b) The western blot images of Pre1 after reactions. c) The western blot images of Glc7 after reactions. d) The western blot images of H2B and H2Bub in both high contrast and low contrast. (Slight leakages of ladder into other lanes can be seen in low-contrast images). e) The intensities of the regions (yellow boxes in a) were determined from images and plotted. The intensities for reacted H2B(product) and un-reacted H2B(leftover) were compared , showing the reaction efficiency is high.

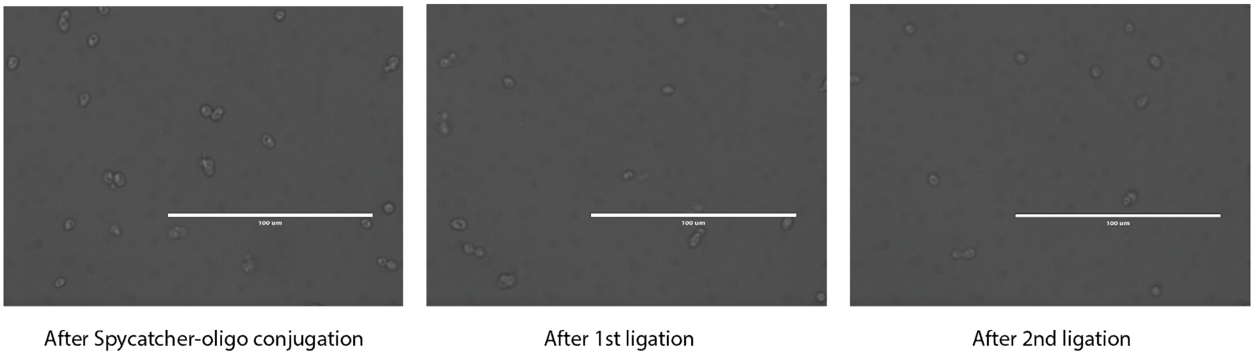

Supplementary Figure 3. Cell morphology under the microscope after spycatcher reaction, first ligation, and second ligation. Individual intact cells can be observed after each step without morphological changes, confirming that each single cell could be used as compartments during ”split-pool” barcoding. (Scale bar: 100um)

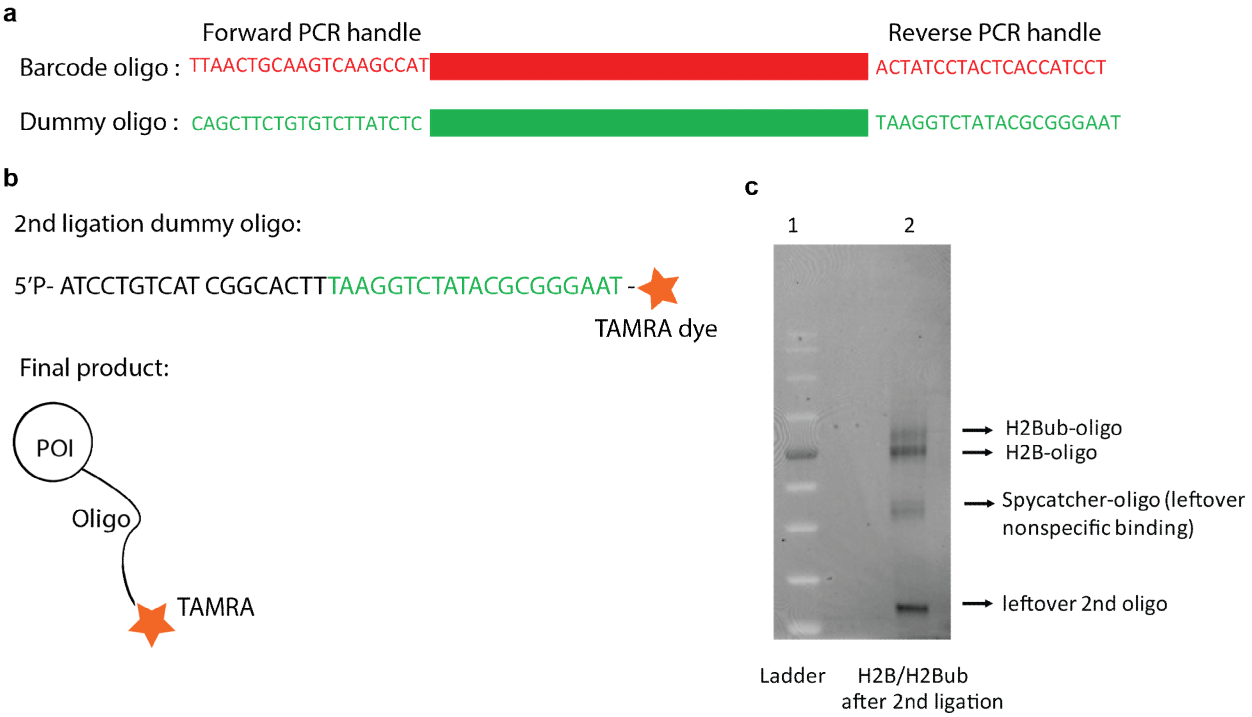

Supplementary Figure 4. Design of dummy oligo. a) The dummy oligo has the same length as the barcode oligo, but has different sequences in the PCR handle. Therefore, proteins labeled with dummy oligo will co-migrate with proteins labeled with barcode oligo during gel electrophoresis, but will not be amplified during library preparation. b) A TAMRA dye is incorporated into the second ligation oligo used in the dummy sample. The protein-oligo conjugate can then be visualized on the gel using fluorescence. c) The fluorescent gel image acquired by a typhoon scanner. H2Bub-oligo and H2B-oligo conjugated with TAMRA dye could be identified. The leftover of 2nd ligation oligo was also found. We also observed a band whose size corresponds to spycatcher-oligo after two rounds of barcodes ligation. This may come from unreacted, non-specifically bound spycatcher-oligos inside the cells that are eventually further barcoded during pool-split barcoding. This spycatcher-full-length oligo product will interfere with quantification if not separated by gel electrophoresis.

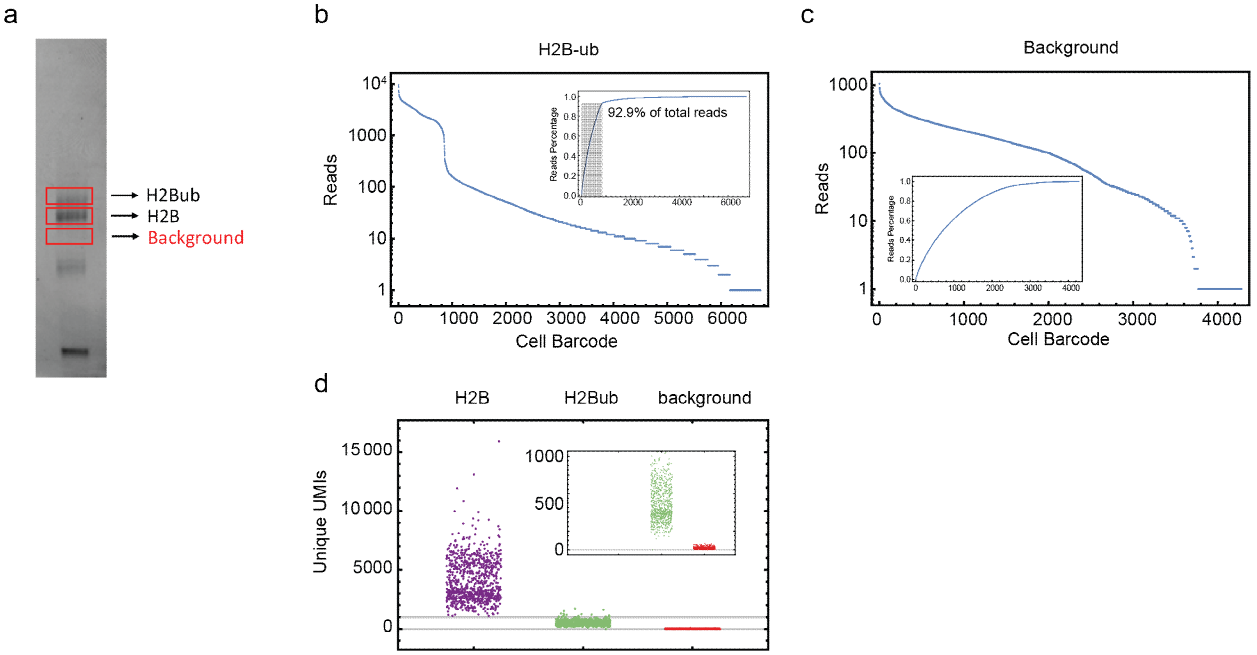

Supplementary Figure 5. Identification of cell barcodes. a) Two bands corresponding to targeted proteins (H2Bub, H2B) and a band corresponding to background were cut from the gel and DNA-protein conjugates were extracted and sequenced by next-generation sequencing. b) The total number of reads per barcode was plotted in the descending order for the H2Bub band (**Figure 2b**). Similar to the H2B band, a clear cut-off can be identified that separates real cell barcodes from spurious barcodes with a low number of reads. The real cell barcodes identified from H2B and from H2Bub are almost the same (848 out of 850), further confirming that those barcodes represent real single cells. c) By contrast, barcodes from the background do not show a clear cutoff. d) The unique UMIs (a.k.a., protein copy numbers) associated with the real barcodes from H2Bub and H2B bands and from the background band. Each dot corresponds to one real cell barcode. This result shows that the gel has a low background.

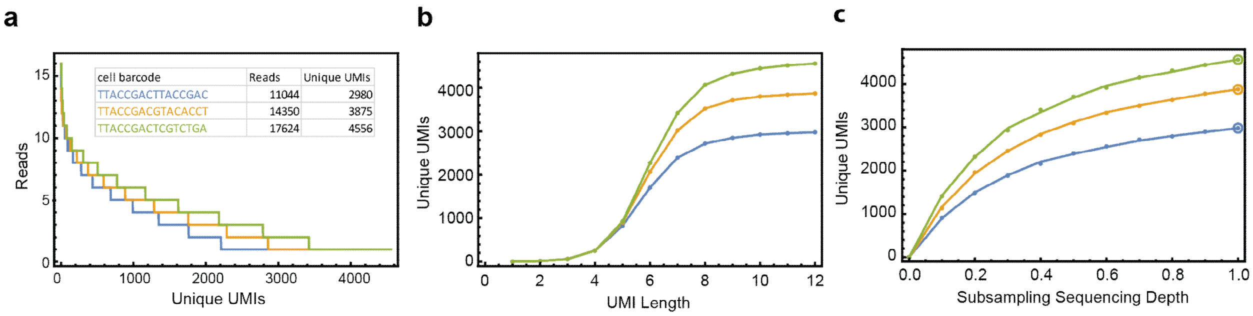

Supplementary Figure 6. Quantification of protein copy numbers by counting UMIs. a) The number of reads for each UMI in 3 example cells. The number of reads associated with UMIs varies from 16 to 1, demonstrating the necessity of using UMI to correct PCR duplication. The inset shows the total reads and unique UMIs associated with each barcode. b) The number of unique UMI identified when sublength of UMIs is taken for 3 example cells. The number of UMIs increased with the length of the UMIs and reached a plateau after around 10nt, indicating that the length of UMI (12nt) have enough coding space to encode all proteins in single cells. c) The number of unique UMIs identified when sequencing depth (the number of total reads) is subsampled. As sequencing depth increases, the number of uniquely identified UMIs increases and reached a plateau at full sequencing depth (1.0), indicating that all the UMIs are

sufficiently sampled. Based on these premises, the protein copy number from single cells can be presented by the number of unique UMIs.

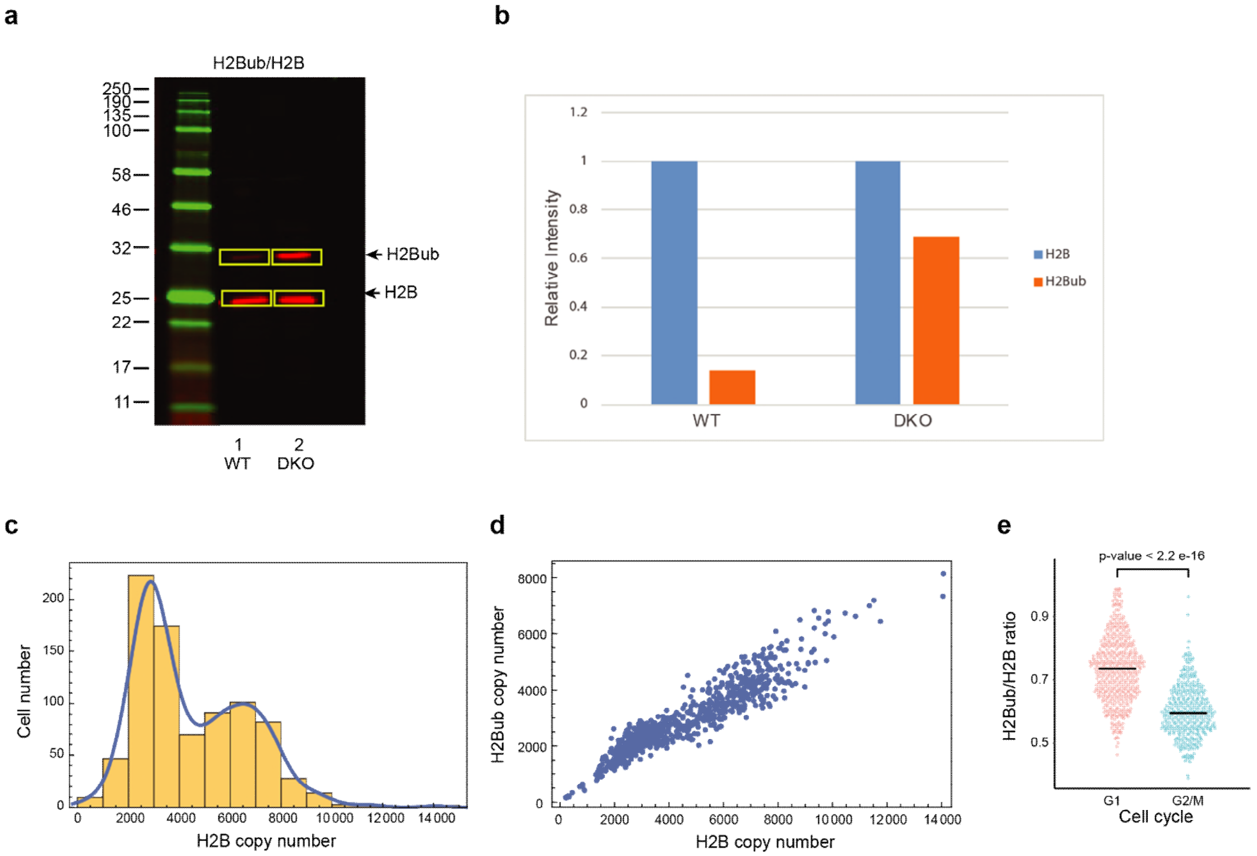

Supplementary Figure 7. Analysis of UBP8 and UBP10 double knock-out strain. a) Western blot images of H2B for wild-type strain (WT) and double knockout strain (DKO). b) The ratio between H2Bub and H2B measured from images. The H2Bub/H2B ratio is ~12% in WT and ~69% in DKO. c) Histogram of H2B copy number in single cells for DKO strain. d) H2B and H2Bub copy number in single cells for DKO strain, each dot corresponding to one single cell. e) The distributions of H2Bub/H2B ratio for cells in G1 and G2/M stages, respectively in dko strain. Similar with wt strain, the two stages have different distributions (Welch's t-test, $n_1 = 361$, $n_2 = 487$).

II References

1. Gingold, E. B. Cloning in Yeast. in Techniques in Molecular Biology: Volume 2 (eds. Walker, J. M. & Gaastra, W.) 140–158 (Springer US, 1987).

2. Bornemann, S., Rietschel, B., Baltruschat, S., Karas, M. & Meyer, B. A novel polyacrylamide gel system for proteomic use offering controllable pore expansion by crosslinker cleavage. *Electrophoresis* **31**, 585–592 (2010).

3. Cusanovich, D. A. *et al.* Multiplex single cell profiling of chromatin accessibility by combinatorial cellular indexing. *Science* **348**, 910–914 (2015).
