# Supplementary material for: Detecting protein and post-translational modifications in single cells with iDentification and qUantification sEparaTion (DUET)
Source: Commun Biol. 2020 Aug 3;3:420. doi: 10.1038/s42003-020-01132-8 (PMC7400673; doi:10.1038/s42003-020-01132-8)
Supplement: Supplementary file 5 — Description of Additional Supplementary Files [file 42003_2020_1132_MOESM5_ESM.pdf]

## Description of Additional Supplementary Files

**Supplementary Data 1**: 1st round T7 ligation and 2nd round T7 ligation barcoding sequence and bridge sequence

Tab_1: The bridge oligo sequences used in $1^{st}$ round T7 ligation and $2^{nd}$ round T7 ligation.
Tab_2: The $1^{st}$ round T7 barcoding oligos (96 of them).
Tab_3: The $2^{nd}$ round T7 barcoding oligos (96 of them).

**Supplementary Data 2**. Collision rate estimation.

The expected collision rate is calculated by assuming a Poisson sampling process. Specifically, the "Zero" column represents the number of cell barcodes that are sampled zero times, which is calculated by $9126 \times (1 - \frac{1}{9216})^n$, where 9216 is the total number of cell barcodes(96*96), and n is the number of cells. The "One" column represented the number of cell barcodes that are sampled one time, which is calculated by $n \times (1 - \frac{1}{9216})^n$. The "Collision" column represents the cell barcodes that are sampled more than once(collision), which is calculated by 9126-Zero-One. The collision rate is then calculated by Collision/(Collision + One). Red color indicates the cell number used in the experiment.

**Supplementary Data 3**: Single-cell protein copy number matrix.

Tab_1: single-cell protein copy numbers for wild-type strain. Each row represents a unique cell barcode (a.k.a a single cell). The first column corresponds to H2B copy number and thesecond column corresponds to H2Bub copy number.
Tab_2: single-cell protein copy numbers for double-knockout strain. Each row represents a unique cell barcode. The first column corresponds to H2B copy number and thesecond column corresponds to H2Bub copy number.
